# Laryngeal Neuroendocrine Tumor With Elevated Serum Calcitonin: A Diagnostic and Therapeutic Challenge. Case Report and Review of Literature

**DOI:** 10.3389/fendo.2020.00397

**Published:** 2020-07-16

**Authors:** Tiziana Feola, Giulia Puliani, Franz Sesti, Roberta Modica, Marco Biffoni, Cira Di Gioia, Raffaella Carletti, Emanuela Anastasi, Valentina Di Vito, Roberta Centello, Andrea Lenzi, Andrea M. Isidori, Antongiulio Faggiano, Elisa Giannetta

**Affiliations:** ^1^Department of Experimental Medicine Sapienza University of Rome, Rome, Italy; ^2^Neuroendocrinology, Neuromed Institute, IRCCS, Pozzilli, Italy; ^3^Department of Clinical Medicine and Surgery, University of Naples Federico II, Naples, Italy; ^4^Department of Surgical Sciences, Sapienza University of Rome, Rome, Italy; ^5^Department of Radiological, Oncological and Pathological Sciences, Sapienza University of Rome, Rome, Italy; ^6^Department of Molecular Medicine, Sapienza University of Rome, Rome, Italy

**Keywords:** larynx, calcium gluconate infusion test, neuroendocrine tumor, everolimus, neck, medullary thyroid carcinoma

## Abstract

**Introduction:** Laryngeal neuroendocrine neoplasms (NENs) are a rare group of NENs of the neck, which commonly show immunostaining for calcitonin. Laryngeal NENs with calcitonin hypersecretion and lymph node metastases represent a diagnostic and therapeutic challenge, which should be included in the differential diagnosis of medullary thyroid carcinoma (MTC). We report a complex case of laryngeal NEN with calcitonin hypersecretion and a review of the literature.

**Case Presentation:** A 59-year-old man presented with dysphagia, dyspnea, and lateral cervical mass; he was a smoker. At first imaging, a laryngeal lesion with lateral cervical lymphadenopathies was found, and it resulted as a moderately differentiated neuroendocrine tumor (G2), Ki67 = 5%, positive for calcitonin. Increased levels of serum calcitonin (50 pg/ml) were found. The patient started somatostatin analogs for lesions positivity to somatostatin receptor-based imaging. After 5 months, the disease progressed at 18F-fluorodeoxyglucose (^18^F-FDG) PET-CT, and also new painful cutaneous lesions occurred. Considering high serum levels of calcitonin, differential diagnosis with MTC was required. Patient performed a thyroid color Doppler ultrasound, nodule fine needle aspiration, calcitonin dosage in fine needle washout fluid, and a calcium gluconate stimulation test. After multidisciplinary evaluation, we decided to perform a total thyroidectomy associated with lateral cervical lymphadenectomy and resection of skin metastases. No MTC was found. Two of the five resected lymph nodes, left upper parathyroid, and skin lesions were metastases of NEN G2, positive for calcitonin. After 2 months, new painful skin lesions occurred, and a target therapy with everolimus 10 mg/day was started. After 6 months of therapy, partial metabolic response with a reduction of 53.7% of radiotracer uptake at primary tumor was detected together with an improvement of patient's quality of life.

**Conclusions:** The present case is the seventh described in the literature of laryngeal NEN associated with elevated serum calcitonin levels and the first case with parathyroid metastasis, suggesting the importance of a correct differential diagnosis between MTC and calcitonin-secreting laryngeal NEN, using an integrated approach of biochemistry and advanced imaging. This is also the first time that somatostatin analogs and then everolimus were used in this setting, resulting in clinical and partial metabolic response.

## Introduction

Laryngeal neuroendocrine neoplasms (NENs) are a rare group of NENs of the neck, divided into epithelial (carcinomas), and neural-type tumors (paraganglioma) (1). Primary epithelial-derived neuroendocrine lesions probably arise from the Kulchitsky cells, neuroendocrine cells identified in the basal and middle layer of the respiratory epithelium, particularly in the ventricle and the subglottis (2). In the WHO Blue Book 2017, the classification of laryngeal NEN has been recategorized as follows: well-differentiated carcinoma G1 (previously classified as carcinoid), very uncommon; moderately differentiated carcinoma G2 (previously called atypical carcinoid), the most frequent type; and poorly differentiated neuroendocrine carcinoma (NEC) G3, including two subtypes, the small cell NEC (SmCNEC) and the large cell NEC (LCNEC) (3). All laryngeal NENs affect men more often than women (3:1), usually in the fifth to seventh decade of life, often with a history of smoking; the most frequent location is the supraglottis (3, 4). Patients typically present with non-specific clinical symptoms related to obstructive mass lesion, like hoarseness, dysphagia, and sore throat. In rare cases, patients present with an aberrant paraneoplastic syndrome due to hormone overproduction by the tumor (1, 5). Local excision is the best treatment, alone for well-differentiated tumors and in combination with elective neck dissection for moderately differentiated ones (4). SmCNEC and LCNEC are aggressive tumors, which take benefit most from chemoradiotherapy (4). Laryngeal NENs typically show neuroendocrine histological and immunohistochemical features, including expression of chromogranin A, synaptophysin, and cytokeratins. Calcitonin can be a further neuroendocrine marker of laryngeal NENs (6). In addition, other NENs [pancreatic NENs (PanNENs) and pheochromocytomas] expressed calcitonin, generally in the presence of inappropriately elevated serum calcitonin levels (7, 8). Particularly, calcitonin was expressed in 10.9% of the cases in a clinical-pathological study of 229 PanNENs, suggesting that calcitonin immunoreactivity is not an exceptional event in PanNENs (9). Surprisingly, although calcitonin immunostaining is common in laryngeal NENs, only six cases with elevated serum calcitonin levels are described in the literature (2, 10–14). Although rare, primary epithelial-derived laryngeal NENs should be included in the differential diagnosis of medullary thyroid carcinoma (MTC), particularly when presenting with elevated serum calcitonin levels and positive cervical lymph node metastases, without any primary tumor within thyroid gland (1). Conversely, in rare cases, MTC can occur also into the larynx, so the differential diagnosis with primary laryngeal NENs is challenging (15). We report a complex case, with peculiar features never described in the literature, of laryngeal NEN with elevated serum calcitonin levels, parathyroid, and painful cutaneous metastases. A review of the literature of laryngeal NENs with calcitonin hypersecretion is also reported.

## Case Presentation

A 59-year-old man attended the endocrinology outpatient clinic for the occurrence of dysphagia, dyspnea, and a left lateral cervical mass, which appeared 2 years before. He had a history of smoking one pack of cigarettes per day since age 18. In the last 7 years, he had a history of hypertension and hepatitis C treated with sofosbuvir and velpatasvir with negativization of viremia 4 years before. The patient had no familial history for neoplasms or endocrine diseases. On clinical examination, left lateral cervical mass was fixed and firm, and no other relevant alterations were found.

In the diagnostic workup, contrast-enhanced computed tomography (CT) was performed and showed the presence of a vascularized lesion of the supraglottic left emilarynx (maximum diameter, 19 mm), which invaded the left epiglottic vallecula, aryepiglottic fold, piriform sinus, and free wall of the epiglottis. CT scan revealed also thickening of the left vocal cord and adjacent soft tissue and the presence of multiple lateral cervical lymphadenopathies, the most evident on the left side (maximum diameter, 16 mm), and one left supraclavear lymphadenopathy (maximum diameter, 17 mm).

At fibrolaryngoscopy, the laryngeal lesion was biopsied, revealing a moderately differentiated neuroendocrine tumor (grade G2), Ki67 index of 5%. Immunohistochemistry was positive for synaptophysin, neuron-specific enolase, chromogranin A, pan-cytokeratin, cytokeratin 19, and calcitonin. Circulating neuroendocrine markers (neuron-specific enolase and chromogranin A) were within the normal range, except for calcitonin (50 pg/ml; normal range, 0–10).

**Figure 1:**
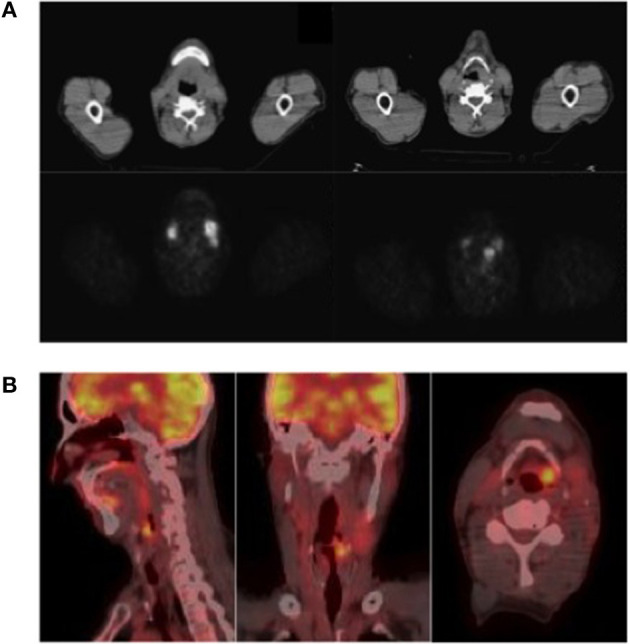
**(A)** 68Ga-DOTATOC PET-CT (upper panel) and **(B)** 18F-FDG PET-CT (lower panel). **(A)** 68Ga-DOTATOC PET-CT showed accumulation of radiotracer in left epiglottic region; **(B)** 18F-FDG PET-CT showed uptake of the radiotracer in the left emilarynx lesion, SUV max 9.3, and in two laterocervical lymph nodes, SUV max 11.4 and 7.

A ^68^Ga-DOTATOC PET-CT showed accumulation of radiotracer in the left epiglottic region (Figure 1A). A somatostatin analog (SSA) therapy was started (octreotide LAR, 30 mg every 28 days). After 5 months from the first morphological imaging, 18F-fluorodeoxyglucose (^18^F-FDG) PET-CT showed uptake in the left epiglottic region [standardized uptake value (SUV) max, 6], but the size and the morphology remained unchanged. On the contrary, the left lateral cervical mass (SUV max, 8) was incremented in dimensions (21 vs. 18 mm). After 2 months, two pericentimetric cutaneous lesions appeared in abdominal and zygomatic regions, confirmed as cutaneous metastases of well-differentiated neuroendocrine tumor at biopsy.

After 9 months of treatment, an ^18^F-FDG PET-CT (Figure 1B) showed increased dimensions (22 vs. 19 mm) and uptake of the radiotracer (SUV max, 9.3 vs. 6) in the left emilarynx lesion. Moreover, the ^18^F-FDG PET-CT showed an increased accumulation in the left lateral cervical lymph node (SUV max, 11.4 vs. 8) and a new focus of uptake in another left lateral cervical lymph node (SUV max, 7; maximum diameter, 10 mm). Clinically, dysphagia worsened, limiting the possibility to eat solid food, and pain became more frequent and severe, requiring oral morphine therapy.

**Figure 2:**
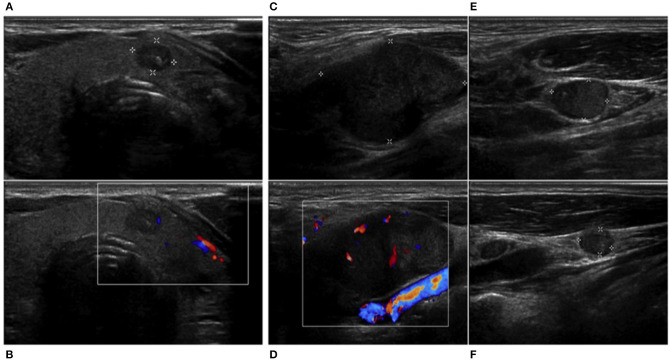
Thyroid and neck ultrasound. An irregular hypoechoic nodule (11 × 7 mm) with **(A,B)** calcifications in the paraisthmic portion of the left thyroid lobe and **(C,E,F)** multiple suspected lateral cervical rounded, hypoechoic, and inhomogeneous lymphadenopathies without ilum, showing an panel **(D)** unorganized peripheral and central vascularization.

Considering high levels of serum calcitonin, differential diagnosis between MTC and calcitonin-secreting laryngeal NEN was required. A thyroid color Doppler ultrasound was performed, and it showed an irregular hypoechoic nodule (11 × 7 mm) with calcifications in the paraisthmic portion of the left thyroid lobe and also confirmed the presence of multiple lateral cervical lymphadenopathies (Figure 2). The nodule was classified as TIR3B by fine needle aspiration (FNA) cytology, according to Italian consensus for the classification and reporting of thyroid cytology (16). Calcitonin in the washout fluid of FNA was negative (2.9 pg/ml) (17). Considering discrepancy between serum calcitonin levels and calcitonin in the washout fluid of FNA, we performed a calcitonin stimulation test. The highest value was 56.6 pg/ml, not diagnostic for MTC. Molecular analysis for RET mutation on peripheral blood cells was negative.

Multidisciplinary evaluation by the Neuroendocrine Tumor task force (NETTARE) and thyroid cancer multidisciplinary unit of our University “Sapienza”–Policlinico Umberto I Hospital proposed total thyroidectomy for cytological diagnosis of TIR3B, associated to cervical lymphadenectomy and cutaneous metastases resection aimed to tumor debulking and pain relief.

**Figure 3:**
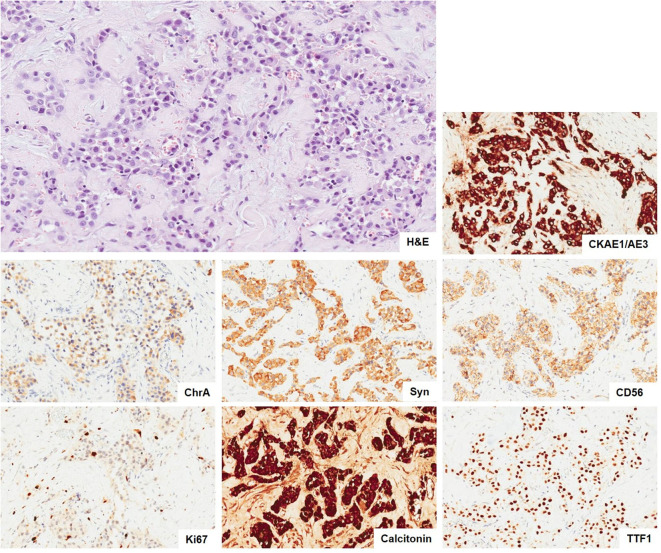
Metastasis of neuroendocrine tumor. The neoplasia infiltrating the connective tissue show a solid and only focal tubuloacinar arrangement of cells with moderate nuclear atypia. There is not any necrosis. (H&E, × 10). The immunohistochemical stains (× 10) show positivity of tumor cells for epithelial (CKAE1/AE3) and neuroendocrine markers (ChrA, Syn, and CD56) and a proliferative index Ki67 of 5%. The tumor cells are also positive for calcitonin and TTF-1. H&E, hematoxylin and eosin; CKAE1/AE3, pan cytokeratins AE1/AE3; ChrA, chromogranin A; Syn, synaptophysin; CD56, NCAM; TTF-1, thyroid transcription factor 1.

The histological examination revealed benign hyperplastic nodules in the thyroid gland, two of the five resected lymph nodes and cutaneous fragments as metastasis of a neuroendocrine tumor G2, positive for calcitonin, as shown in Figure 3. Interestingly, left superior parathyroid gland was the site of 1 mm metastasis.

After surgical intervention, the patient reported rapid pain relief, and oral morphine therapy was withdrawn. Unfortunately, after 2 months, disease progressed under SSA therapy with the appearance of three new cutaneous metastases. Therefore, we decided to start everolimus, with a starting dose of 10 mg/day. After 3 weeks of therapy, the patient developed oral mucositis (grade G3), characterized by pain and nutritional impairment, which required treatment withdrawal. After appropriate antifungal therapy, everolimus treatment was restarted at the half dose of 5 mg, then increased to 10 mg with good tolerance and safety profile. After 6 months from the beginning of the target therapy, ^18^F-FDG PET-CT showed a reduction of 53.8% of the radiotracer uptake in the left emilarynx lesion (SUV max, 4.3 vs. 9.3) and borderline uptake at sternal body (SUV max, 3.2) and at left iliac fossa (SUV max, 2.6).

## Methods

We performed calcitonin stimulation test obtaining multiple blood samples to evaluate calcitonin levels before and after 1, 2, 3, 5, 10, and 15 min after calcium gluconate infusion (25 mg/kg) (18). Serum calcitonin was measured with a commercially available kit (Scantibodies Laboratory, Inc, Santee, CA, USA). The Scantibodies Calcitonin Immunoassay is a two-site immunoradiometric assay (IRMA) for the measurement of calcitonin. Two different goat antibodies to human calcitonin have been purified by affinity chromatography to be specific for well-defined regions of calcitonin molecules. The detection range was between 10 and 1,000 pg/ml, with intra-assay coefficient of variation (CV) of <10%, interassay CV of <9%, and a cutoff value of 10 pg/ml (CV <10%) based on the 95% confidence interval according to manufacturer's specifications. All assays were performed in duplicate and according to the manufacturers' instructions.

All the tissue specimens (thyroid, lymph nodes, and skin) were fixed with 10% formalin and embedded in paraffin. Hematoxylin–eosin (H&E)-stained sections (3 μm) were used for light microscopic examination. Immunohistochemical stains were performed by BOND-III automated IHC stainer (Leica Biosystems, Milan, Italy) with the following BOND ready-to-use antibodies (Novocastra, Newcastle upon Tyne, UK): pan cytokeratins CKAE1/AE3, chromogranin A, synaptophysin, CD56, CT, TTF-1, CEA, PAX8, and Ki67, using HRP-DAB detection system. Immunostaining slide with Ki67 was captured with Aperio scanner (Leica Biosystems, Milan, Italy), and the nuclear positivity (in 500 tumor cells) was quantified manually by a pathologist on digital images and expressed as percentage.

## Discussion

We report a complex case of laryngeal NEN with many peculiar diagnostic and therapeutic features. The importance and peculiarity of the present case are represented by the following points: (1) the rarity of the coexistence of the laryngeal NEN and elevated serum calcitonin levels, although calcitonin immunostaining is common in laryngeal NENs; (2) the first case in which parathyroid metastasis is reported together with the most common lymph nodes and painful cutaneous metastases; (3) the first case in which SSAs and everolimus were used in a well-moderate differentiated laryngeal NEN with clinical and partial metabolic response; (4) the need of a correct differential diagnosis between MTC and calcitonin-secreting laryngeal NEN, which should include dosage of calcitonin in the washout fluid of FNA and calcium gluconate stimulation test, combined with advanced imaging.

Laryngeal NENs typically show neuroendocrine histological and immunohistochemical features including expression of chromogranin A, synaptophysin, and cytokeratins and can stain positive for calcitonin. Rare cases of MTC can extend into the larynx and must be distinguished from primary laryngeal NEN (1). Embryologically, the epiglottis, body of the larynx, and superior parathyroids originated from the mesenchyme of the fourth and the sixth pairs of pharyngeal arches, in which the neural crest cells migrate before developing the characteristics of C cells. Therefore, calcitonin and CEA are both expressed by thyroid C cells and laryngeal neuroendocrine cells, belonging both to the neuroendocrine system (12). To the best of our knowledge, although calcitonin immunostaining is common in laryngeal NENs, only few patients with associated calcitonin hypersecretion were reported (2, 10–14), as summarized in Table 1.

**Table 1:** Summary of case reports of laryngeal neuroendocrine neoplasms (NENs) with elevated serum levels of calcitonin.

**References**	**Age, sex**	**bCT and sCT at diagnosis**	**Location of tumor**	**Treatment**
Our case	59, M	bCT: 50 pg/ml (<10 pg/ml), sCT: 56.6 pg/ml (10 min after calcium gluconate test)	Epiglottis, 2 cervical lymph nodes, skin	TT, lymph node dissection, skin metastases excision, OCT 30 mg/28 days and Everolimus 10 mg/day
(11)	57, M	bCT: 157 pg/ml (0–8 pg/ml)	Right arytenoid, 7 cervical lymph nodes, and thyroid	Total laryngectomy, bilateral neck dissection, and TT
(2)	57, M	bCT: 599 pg/ml (reference range NA)	Epiglottis, cervical lymph nodes, skin nodules in the right arm, bones	Supraglottic laryngectomy, neck dissection, CHT
(12)	58, M	bCT: 48.9 pg/ml (<10 pg/ml)	Right aryepiglottic fold, six subcutaneous pre-laryngeal nodules, inner lower quadrant of left breast	Aryepiglottic cartilage resection, subcutaneous nodules excision
(10)	69, M	bCT: 970 pg/ml (<300 pg/ml)	Right arytenoid	Partial laryngectomy, RT, subtotal T
(13)	55, M	bCT: 3,790 pg/l (<100 pg/l), sCT: 6,378 pg/ml (5 min after pentagastrin administration)	Epiglottis, three submandibular lymph nodes, skin, brain	RT, CHT, submandibular lymph node resection, left neck dissection, total laryngectomy, skin, and cerebral metastases excision
(14)	54, M	bCT: 1,200 ng/L (<200 ng/l), sCT: 1,500 ng/l (3 min after pentagastrin administration)	Left arytenoid, three cervical lymph nodes	Laryngothyroidectomy

*bCT, basal calcitonin level; sCT, stimulated calcitonin level; TT, total thyroidectomy; OCT, octreotide; T, thyroidectomy; CHT, chemotherapy; RT, radiotherapy*.

Laryngeal NENs commonly affect men more often than women (3:1), usually in the fifth to seventh decade of life, often with a history of smoking; the most frequent location is the supraglottis (3, 4). Patients typically present with non-specific clinical symptoms related to obstructive mass lesion, like hoarseness, dysphagia, and sore throat. Patients generally present with neck lymph nodes metastases, which can appear as cervical mass and with painful cutaneous metastases. In rare cases, patients present with an aberrant paraneoplastic syndrome due to hormone overproduction by the tumor (1, 5). In 1981, Sweeney et al. (14) reported the first case of a laryngeal NEN found to secrete calcitonin in the absence of a primary thyroid tumor. LaBryer et al. (11) described a case report of a primary calcitonin-secreting laryngeal NEN associated with lymph nodes and subcutaneous and thyroid metastases. In the study of Chung et al. in which six patients with laryngeal NEN were analyzed, one patient showed elevated serum calcitonin levels in the late stage of the disease. He developed not only painful skin nodules but also bone metastases (2). Machens et al. described the same phenomenon in a 58-year-old patient. The authors supposed that the cause of the pain in these patients might be related to the excess of secretion of calcitonin or other substances produced by tumor cells (2). Metastases to the skin or scalp, with or without hormone secretion, are a peculiar clinical manifestation of laryngeal NENs, described in up to 22% of the patients (12). Accordingly, skin was the main site of tumor metastasis in our patient. Finally, other two cases of metastasizing moderately differentiated laryngeal NENs that shared many similarities with MTC were described in the literature (10, 13). Calcitonin may therefore be regarded not only as a marker of MTC but also of well to moderately differentiated laryngeal NENs as well as of NENs of other sites (7).

The finding of calcitonin levels higher than the normal range may require a confirmatory stimulation test, which can be useful to identify the possible coexistence of non-thyroidal NENs. In fact, calcitonin-secreting NENs can be distinguished from a C-cell disease by the absence of response to the stimulation test (19); however, specific cutoff for stimulated calcitonin in this setting is missing in the literature. As a general remark, stimulation test has relevance to exclude MTC in an unaffected individual with basal calcitonin in the gray zone, as occurred in NENs (19). Two authors used a pentagastrin stimulation test; the first observed a poor response, while the second, a calcitonin doubling (3,790 pg/ml basal calcitonin vs. 6,378 pg/ml after 5 min of pentagastrin stimulation) (13, 14). The pentagastrin was used for several years for diagnostic purposes, but its unavailability in most countries raised the need to standardize other stimulation test (19). In our case, in the presence of thyroid nodules and borderline serum calcitonin levels, we performed FNA associated with calcitonin dosage in the washout fluid of FNA and a calcium gluconate stimulation test. FNA diagnosed a TIR3B nodule, and calcitonin levels in the washout fluid of FNA were negative with a value of 2.9 pg/ml (17). A systematic review of recently published studies has demonstrated that almost all MTC lesions can be correctly detected by measurement of calcitonin in the washout fluid of FNA (20). Based on this review, and according to American Thyroid Association (ATA) guidelines, FNA calcitonin should be used in diagnostic workup of MTC, with the discretion of the physician (21). In our case, serum calcitonin after calcium stimulation showed a poor response (the highest value was 56.6 pg/ml). The calcium stimulation test has been recently reevaluated in healthy subjects (22), in patients with multinodular goiter and with either familial or sporadic MTC (23), and in thyroid conditions other than MTC (24). Gender-specific cutoffs of basal and stimulating calcitonin for MTC diagnosis were identified in a large series of patients. The best thresholds were >26 and >68 pg/ml for basal calcitonin and >79 and >544 pg/ml for stimulated calcitonin, respectively, in women and men (19). Moreover, in our case genetic screening for RET was performed and resulted negative. All these results suggested the non-thyroidal origin of calcitonin production.

In the literature, the most cases of moderately differentiated laryngeal NENs were treated by radical surgery and chemoradiotherapy (4, 25). In our case, after a multidisciplinary evaluation, we choose to not perform a laryngectomy and tracheostomy considering the young age of the patient, his good performance status, and his preference. We proposed a total thyroidectomy for the indeterminate cytological diagnosis of TIR3B, suspecting a differentiated thyroid carcinoma, associated to cervical lymphadenectomy and cutaneous metastasis resection aimed to NEN debulking and pain relief.

The histological examination revealed benign hyperplastic nodules in the thyroid gland; no MTC was found, and confirmed lymph nodes and cutaneous metastases of NET G2, positive for calcitonin, and interestingly, showed a metastasis in the left superior parathyroid gland.

In view of the positive uptake of Gallium peptides, indicating a neoplasm with high expression of somatostatin receptors, a first-line SSA therapy was started. No other cases of laryngeal NEN treated with SSA are reported, so it represents an unexplored medical treatment (25). In our case, after 9 months of SSA treatment, the disease progressed, so a second-line therapy with everolimus in combination with SSAs was indicated. Considering the recent evidence of sensitivity of well-differentiated lung NEN to mechanistic target of rapamycin (mTOR) inhibitors (26) and the histological similarities between lung and laryngeal NENs, this therapy was proposed to the patient.

## Conclusions

Laryngeal NENs are rare neoplasms, which often represent a diagnostic and therapeutic challenge. We report a complex and peculiar case of a 59-year-old man, with a long history of smoking, affected by laryngeal NEN metastatic to nodes, skin, and parathyroid with elevated serum calcitonin levels. This case underlines the importance of a correct differential diagnosis between intrathyroidal or metastatic (laryngeal) MTC and calcitonin-secreting laryngeal NEN, which should include the measurement of calcitonin in the washout fluid of FNA and a calcium gluconate stimulation test. The management of this case required an integrated approach of biochemistry (basal and dynamic dosages) and advanced imaging (ultrasound, CT, two different PET-CT radiotracers), allowing an absolute diagnostic completeness. The review of the literature showed only other six cases of calcitonin-secreting laryngeal NEN, often metastasizing to nodes and with painful cutaneous metastases, usually treated by radical surgery and/or chemotherapy. This is the first time, to the best of our knowledge, that the SSA treatment and then a targeted therapy with the mTOR inhibitor everolimus were used in laryngeal NEN, resulting in clinical and partial metabolic response with a reduction of 53.7% of radiotracer uptake at primary tumor. However, further studies are needed to confirm the suitability of this therapy and long-term outcomes.

## Data Availability Statement

The original contributions presented in the study are included in the article/supplementary materials, further inquiries can be directed to the corresponding author/s.

## Ethics Statement

Written informed consent was obtained from the individual(s) for the publication of any potentially identifiable images or data included in this article.

## Author Contributions

TF is the first author for this case report and she contributed to the concept and design for the study. EG is the corresponding author supervising this work. AF, GP, FS, and RM contributed to the manuscript preparation. EA contributed to the Methods section in relation to laboratory dosage techniques. CG and RCa contributed to the tissue specimens (thyroid, lymph nodes, and skin) analyses, immunohistochemical stains, and related photos, as well as to the drafting of anatomopathological methods. All authors contributed to manuscript revision and read and approved the submitted version.

## Conflict of Interest

The authors declare that the research was conducted in the absence of any commercial or financial relationships that could be construed as a potential conflict of interest.
